# Supplementary material for: Sex Differences in Cancer Functional Genomics: Gene Dependency and Drug Sensitivity
Source: bioRxiv. 2025 Mar 21:2025.02.05.636540. Originally published 2025 Feb 8. Preprint. [Version 2] doi: 10.1101/2025.02.05.636540 (PMC11838570; doi:10.1101/2025.02.05.636540)

Supplementary Figure S1

A

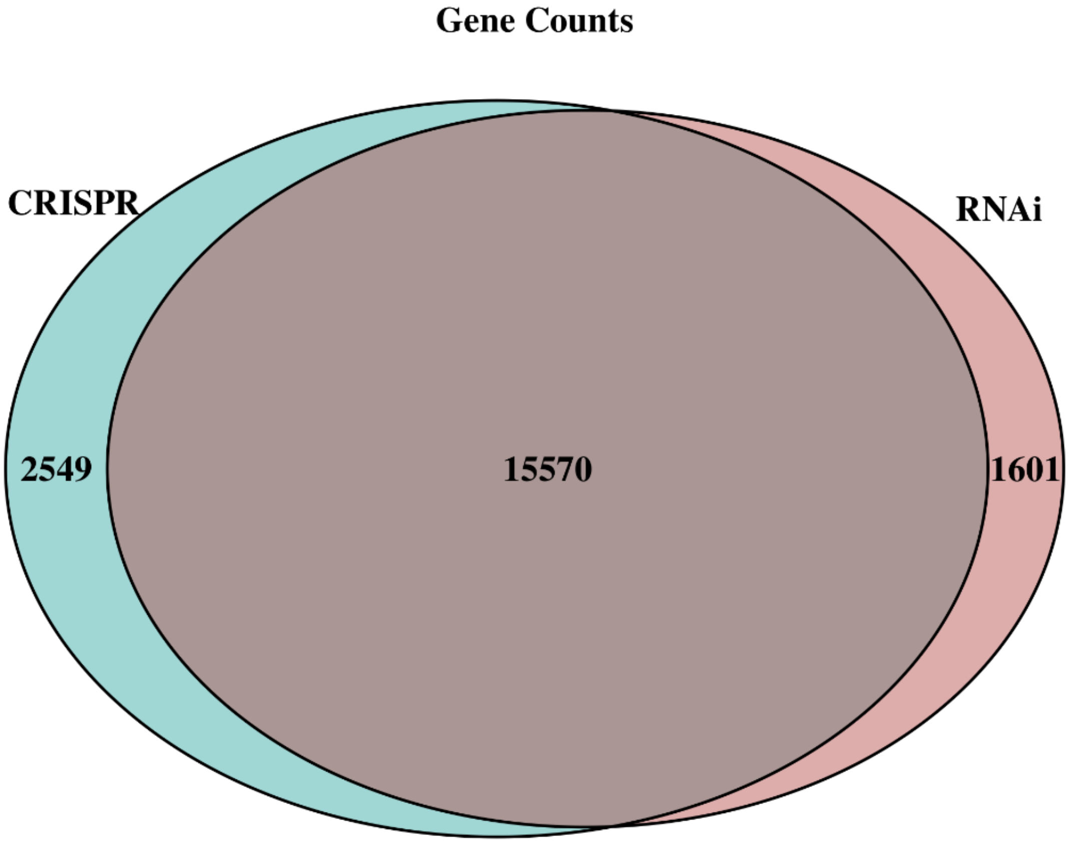

B

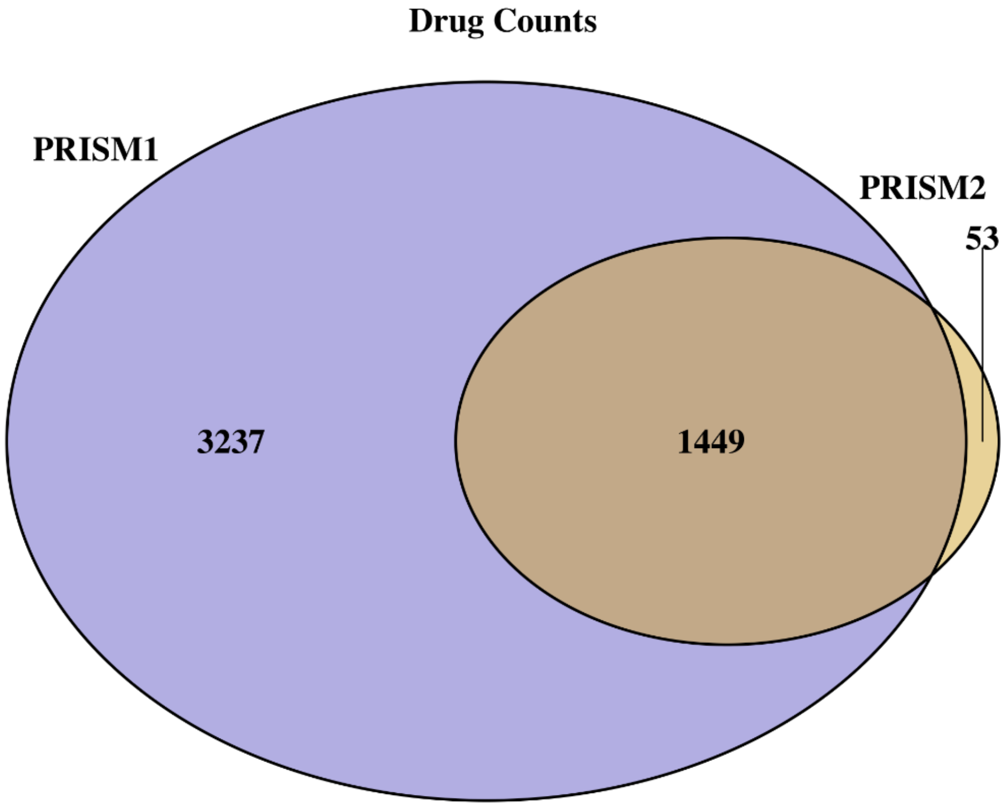

Supplementary Figure S2

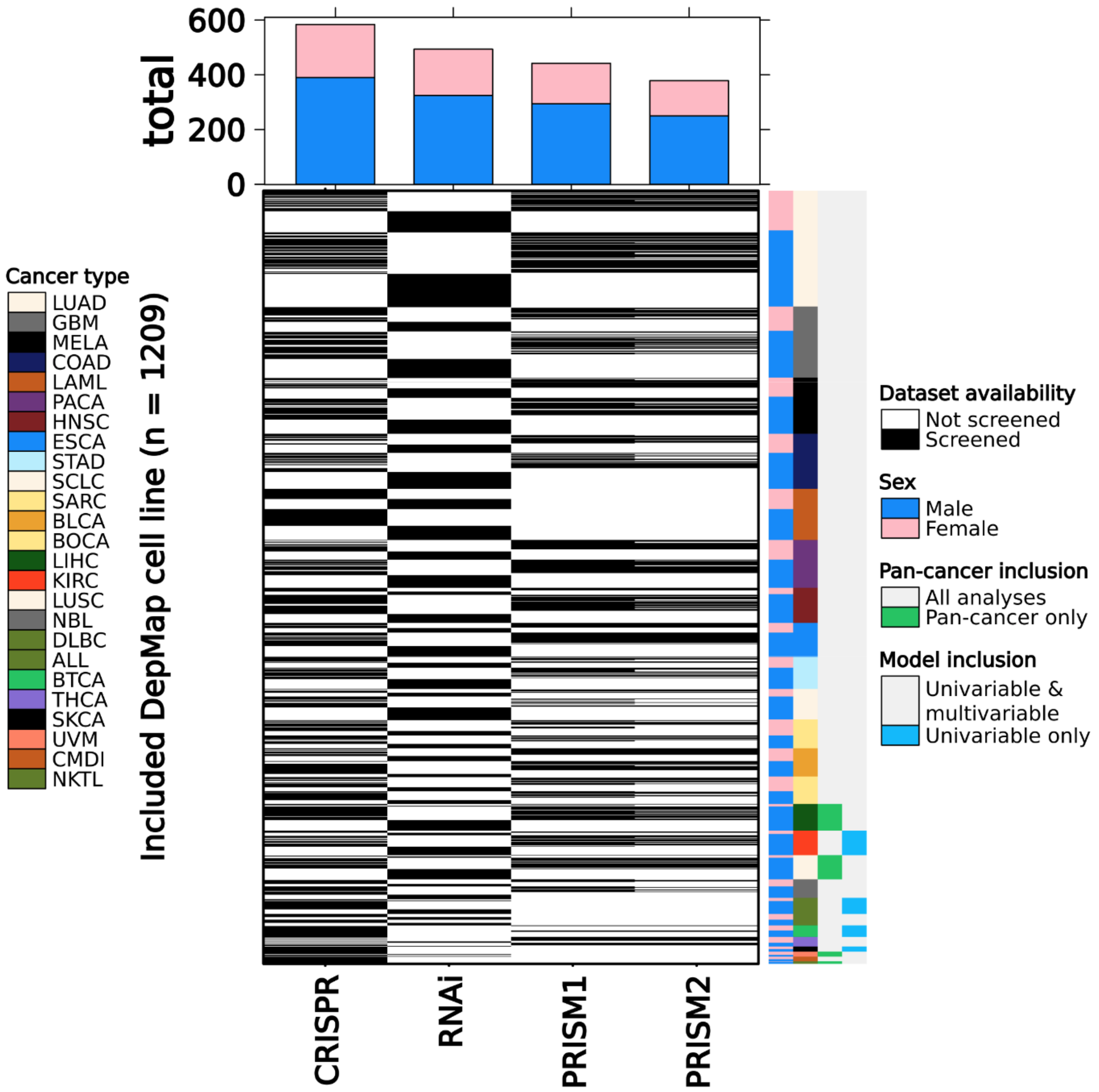

Supplementary Figure S3

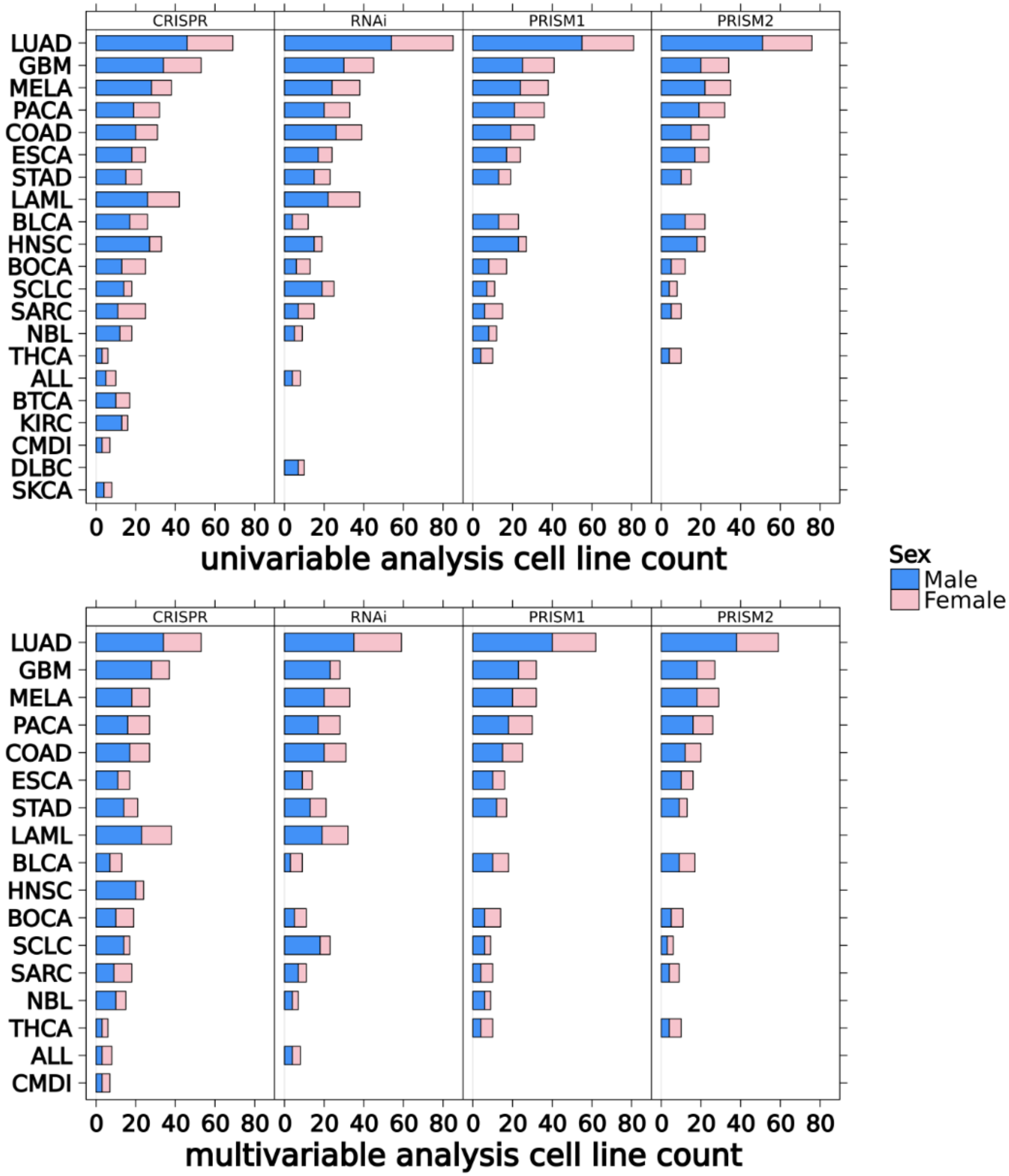

Supplementary Figure S4

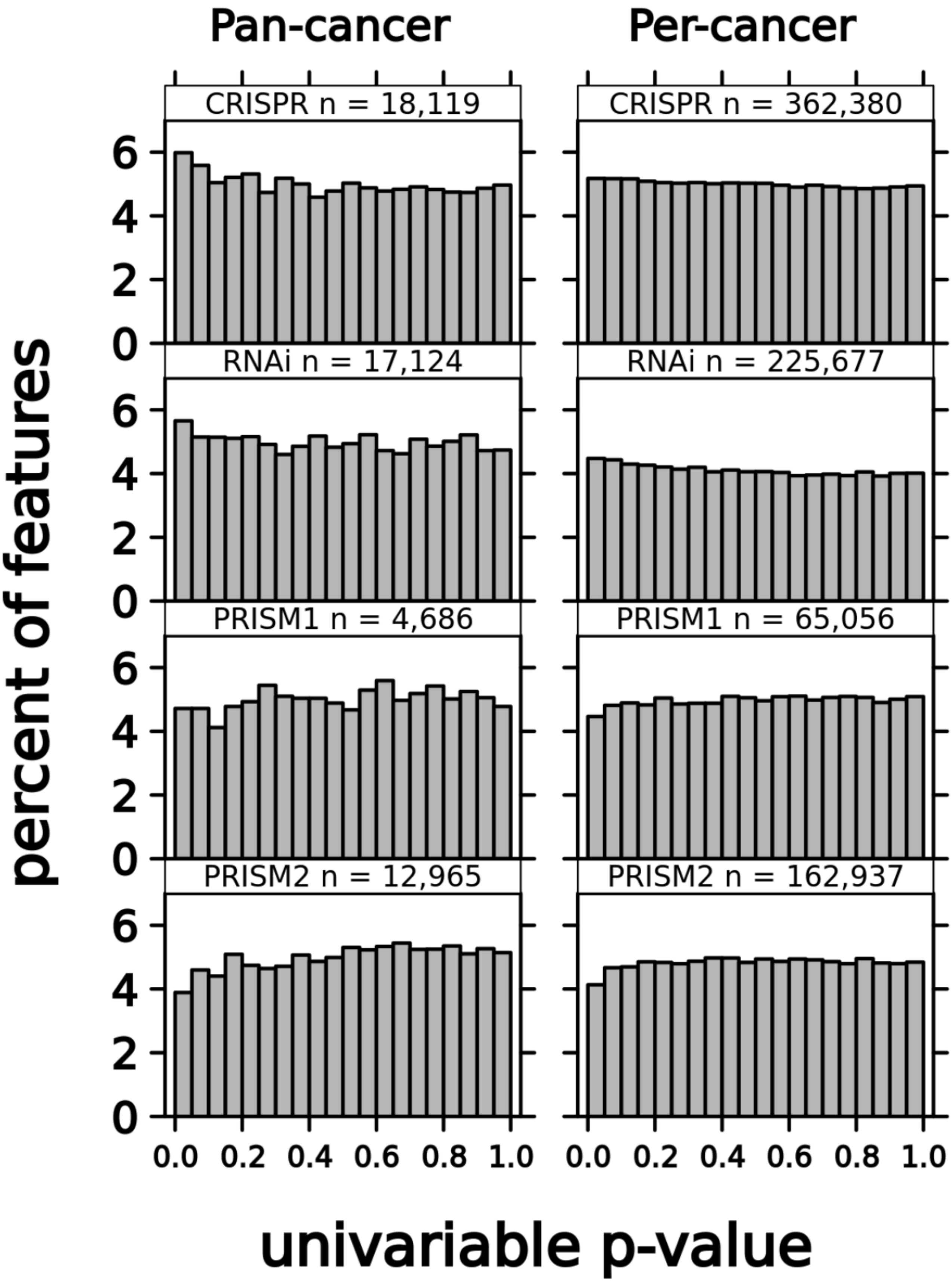

Supplementary Figure S5

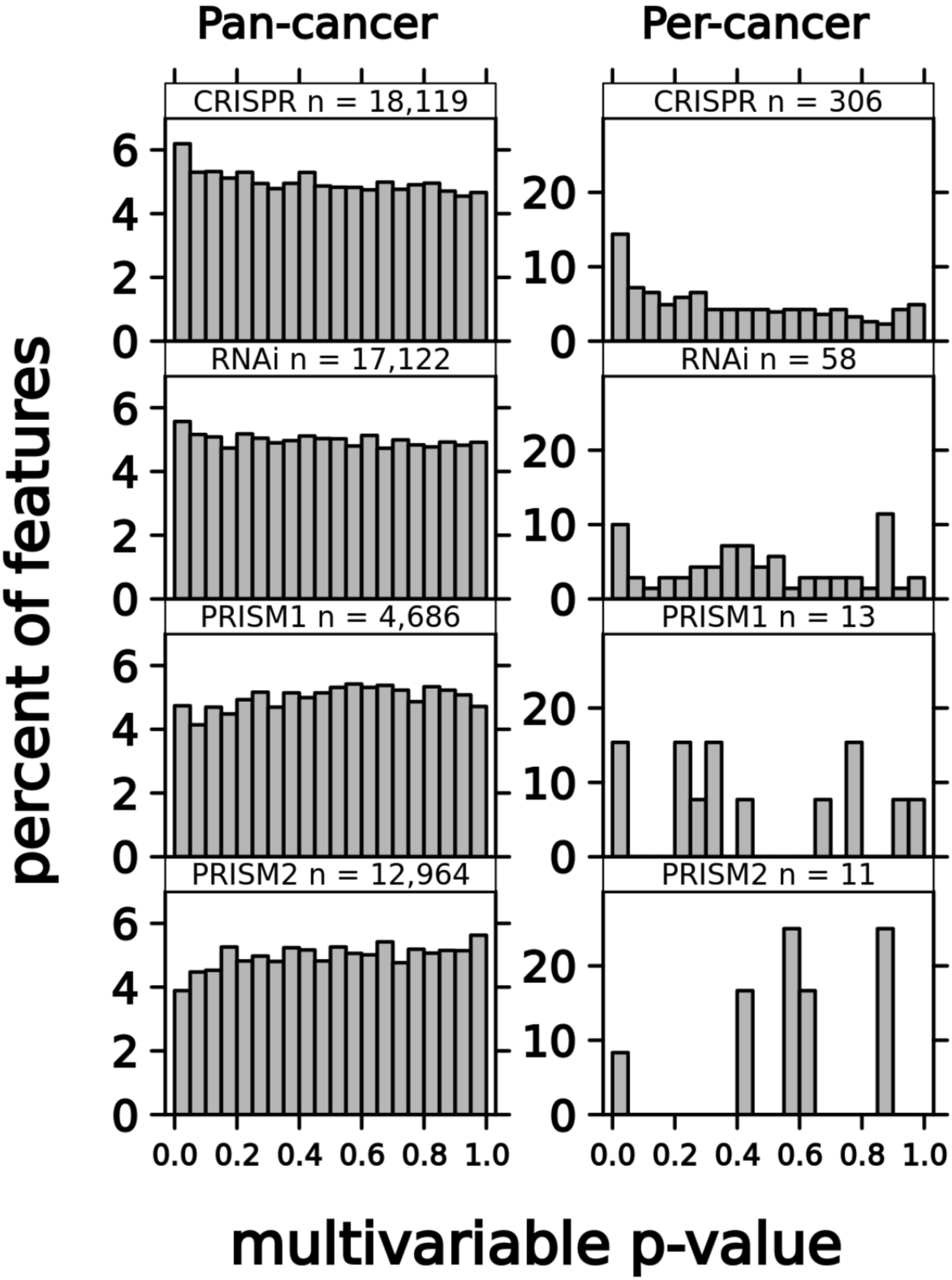

Supplementary Figure S6

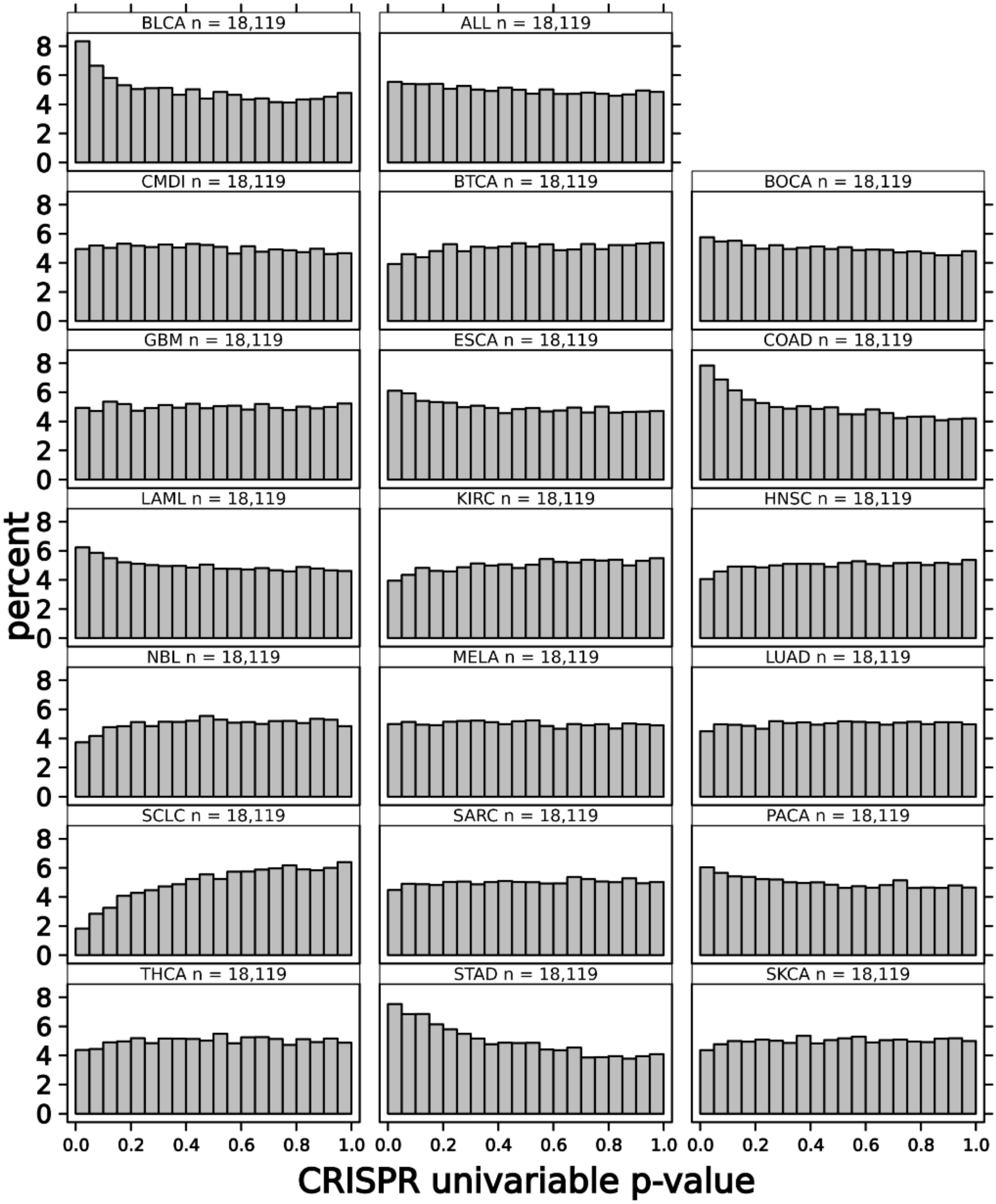

Supplementary Figure S7

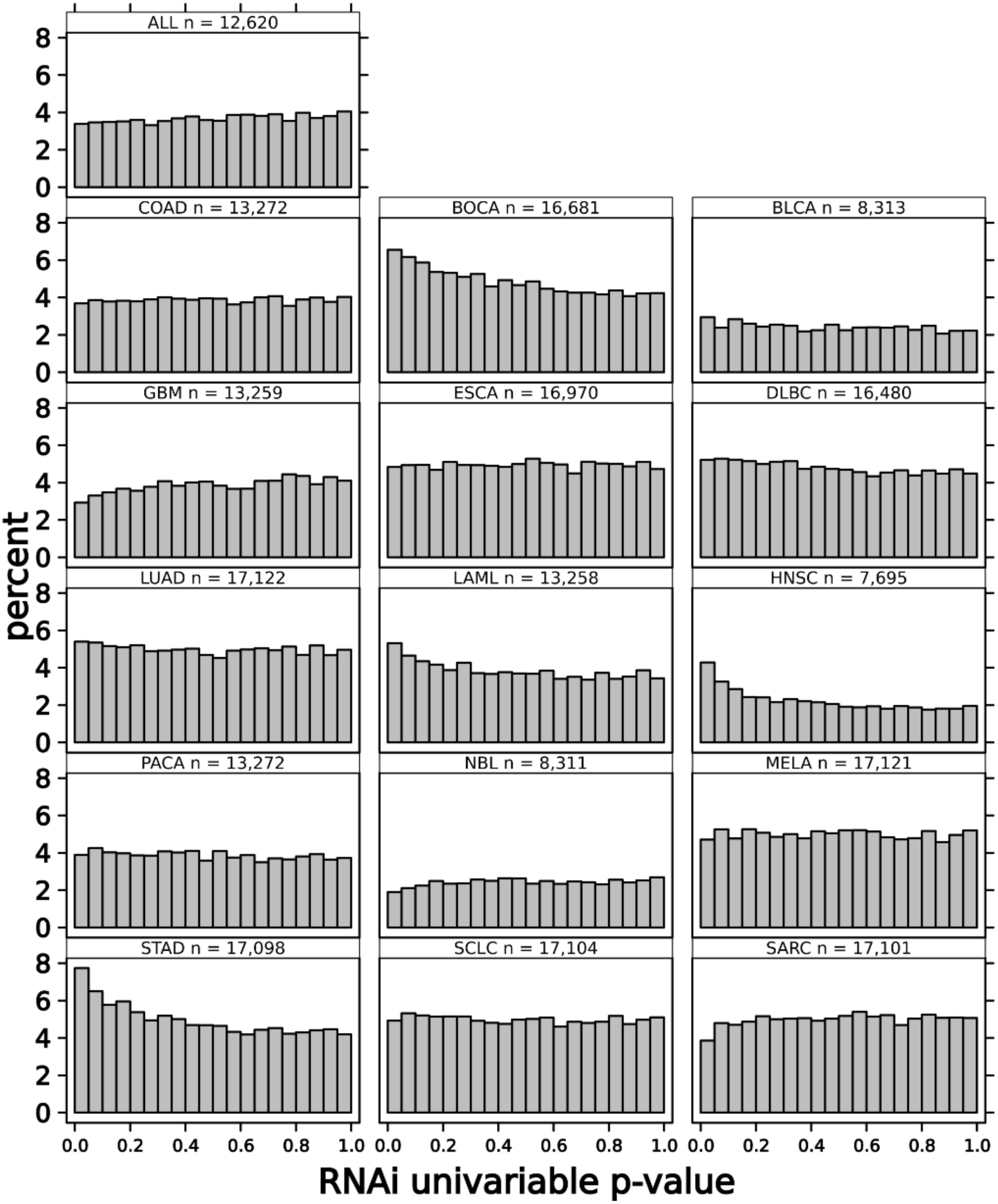

Supplementary Figure S8

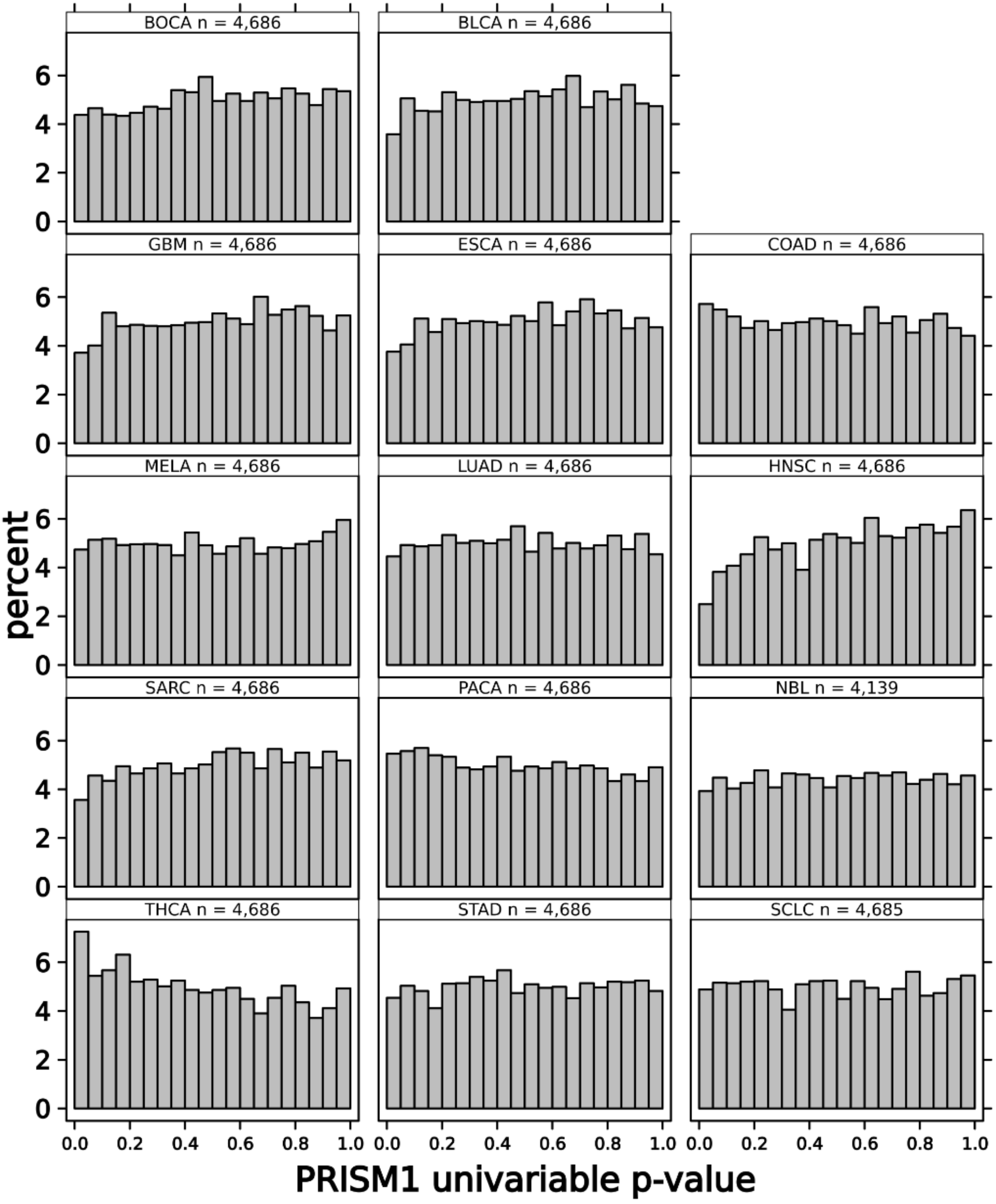

Supplementary Figure S9

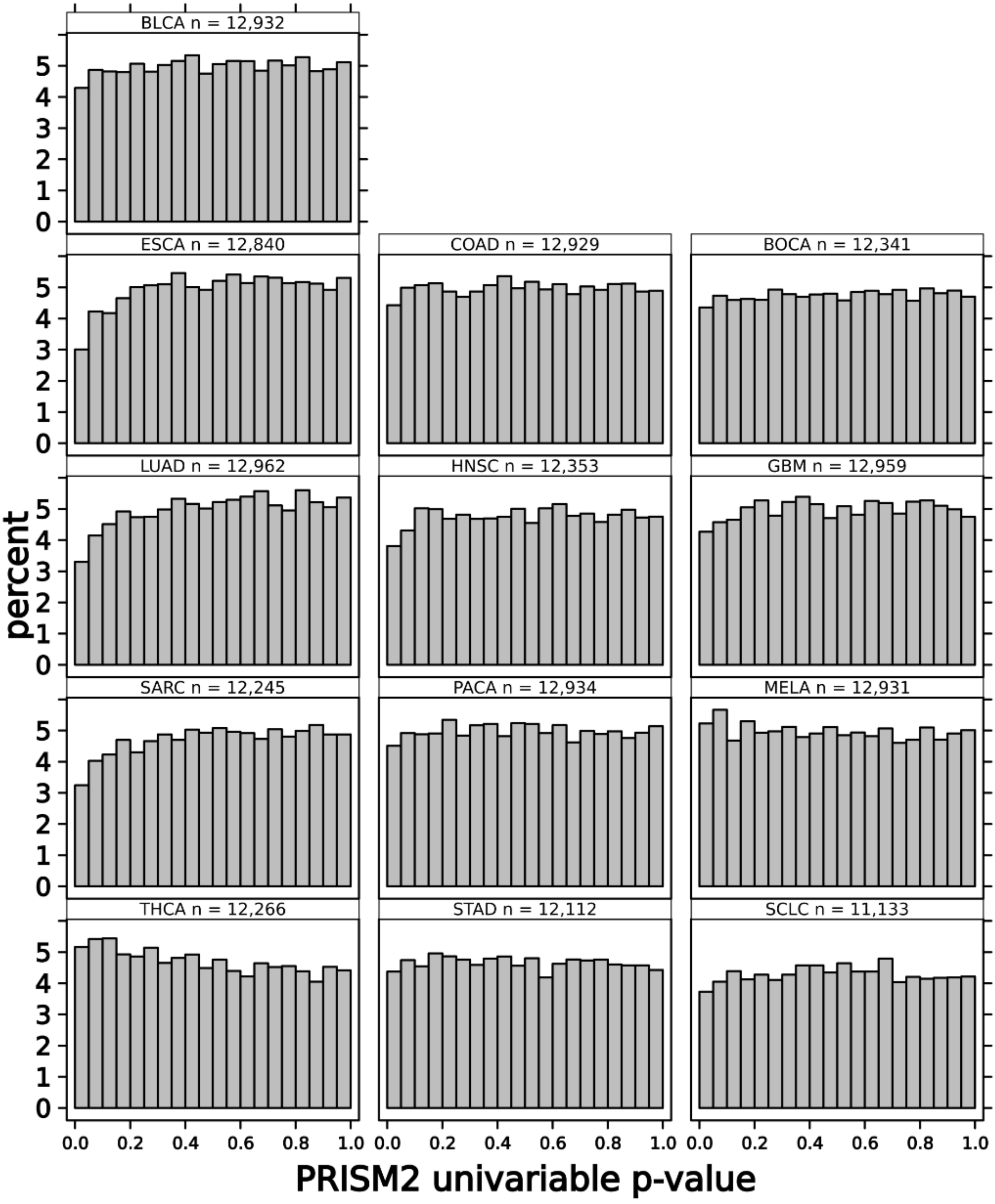

Supplement: Supplement 1 — Supplementary Figure S1. A. Venn diagram of counts of unique autosomal & X-linked genes analyzed for sex differences in gene essentiality between CRISPR knockout and RNAi knockdown screens in the DepMap database. B. Venn diagram of counts of unique drugs analyzed for sex differences in drug sensitivity between PRISM1 primary screens (all drugs at the same concentration), and PRISM2 secondary screens (each drug was screened at eight concentrations, drugs screened multiple times are counted once in this figure). Supplementary Figure S2. Summary of 1,209 cell lines in the DepMap database derived from cancer types included in pan- or per-cancer analyses of sex differences in gene essentiality and drug sensitivity. Cell lines belonging to sex-organ specific cancer types or cancer types without at least one cell line per sex category were excluded from all analyses and are not shown here. Central heatmap indicates included cell line availability by source screen dataset. Not all cell lines are screened in all datasets. Covariate bars (right) indicate cell line sex, cancer type, inclusion in pan-/per-cancer models and univariable/multivariable models. Top panel displays cell line totals by sex for each dataset. Supplementary Figure S3. Counts of cell lines by cancer type included in per-cancer univariable (top) and per-cancer multivariable (bottom) analyses in each source screen dataset. Cancer types with fewer than three cell lines in each sex category are excluded. Supplementary Figure S4. P-value distributions from univariable linear model analyses associating sex with CRISPR knockout induced gene dependency (top), RNA interference knockdown induced gene dependency (middle) and drug screen induced sensitivity (bottom) across pan-cancer analyses (left) and in each cancer individually (right). n = number of tests. Drugs screened at multiple concentrations were tested independently. Supplementary Figure S5. P-value distributions from multivariable linear model analyses ass [file media-1.pdf]
